# Deep learning in interstitial lung disease—how long until daily practice

**DOI:** 10.1007/s00330-020-06986-4

**Published:** 2020-06-14

**Authors:** Ana Adriana Trusculescu, Diana Manolescu, Emanuela Tudorache, Cristian Oancea

**Affiliations:** 1 Department of Pulmonology, University of Medicine and Pharmacy “Victor Babes”, Timisoara, Romania; 2 Department of Radiology, University of Medicine and Pharmacy “Victor Babes”, Eftimie Murgu Square, Number 2, Timisoara, Romania

**Keywords:** Interstitial lung disease, Idiopathic pulmonary fibrosis, Deep learning

## Abstract

Interstitial lung diseases are a diverse group of disorders that involve inflammation and fibrosis of interstitium, with clinical, radiological, and pathological overlapping features. These are an important cause of morbidity and mortality among lung diseases. This review describes computer-aided diagnosis systems centered on deep learning approaches that improve the diagnostic of interstitial lung diseases. We highlighted the challenges and the implementation of important daily practice, especially in the early diagnosis of idiopathic pulmonary fibrosis (IPF). Developing a convolutional neuronal network (CNN) that could be deployed on any computer station and be accessible to non-academic centers is the next frontier that needs to be crossed. In the future, early diagnosis of IPF should be possible. CNN might not only spare the human resources but also will reduce the costs spent on all the social and healthcare aspects of this deadly disease.

**Key Points**

*• Deep learning algorithms are used in pattern recognition of different interstitial lung diseases.*

*• High-resolution computed tomography plays a central role in the diagnosis and in the management of all interstitial lung diseases, especially fibrotic lung disease.*

*• Developing an accessible algorithm that could be deployed on any computer station and be used in non-academic centers is the next frontier in the early diagnosis of idiopathic pulmonary fibrosis.*

**Electronic supplementary material:**

The online version of this article (10.1007/s00330-020-06986-4) contains supplementary material, which is available to authorized users.

## Introduction

Interstitial lung diseases (ILDs) denote over 200 diverse lung disorders that involve inflammation and fibrosis of interstitium, with overlapping clinical, radiological, and pathological features, representing an important morbidity and mortality cause [1].

High-resolution computed tomography (HRCT) is the main method in ILD diagnosis, due to the lung tissue–specific radiation attenuation properties and maximum spatial resolution. The imaging data are evaluated by various textural patterns in the lung window extent and distribution. The assessment focuses on the image’s gray tones and geometrical structures, effectively a repetitive pattern matching problem, creating the perfect context for using computer-aided diagnosis (CAD) systems.

CAD enables medical practitioners to understand and utilize various imagistic investigations, with the help of information technology (IT) techniques [2]. The aim is to improve the diagnosis time and accuracy, with IT acting as support or even as an independent diagnostic option [3]. The CAD algorithms belong to artificial intelligence (AI), emulating human thinking [4]. The ILD diagnosis is essentially an algorithm with the following workflow: in comprehensive history, if physical examination findings and paraclinical investigations (chest X-ray, measurements of lung function, usual and specific blood tests) present a suspicion for ILD, a HRCT is performed [5]. The human factor intervenes next, by verifying the resulted data validity and, if no problems/artifacts are found, searching for patterns in specific locations. If there are clear findings, a diagnosis can be formulated, but if the results are inconclusive, the outcome is a list of possibilities requiring further discussions and more complex, invasive investigations. The individual performing this algorithms is therefore critical to accurate and speedy diagnosis, injecting an overall intrinsic variability. Since CAD can emulate the algorithm, it would make it an ideal choice for this stage, eliminating variances.

This paper aims to offer an in-depth analysis of the way virtual AI improves ILD diagnosis, with an emphasis on convolutional neural networks (CNNs).

## Computer-aided diagnosis history

**Fig. 1:**
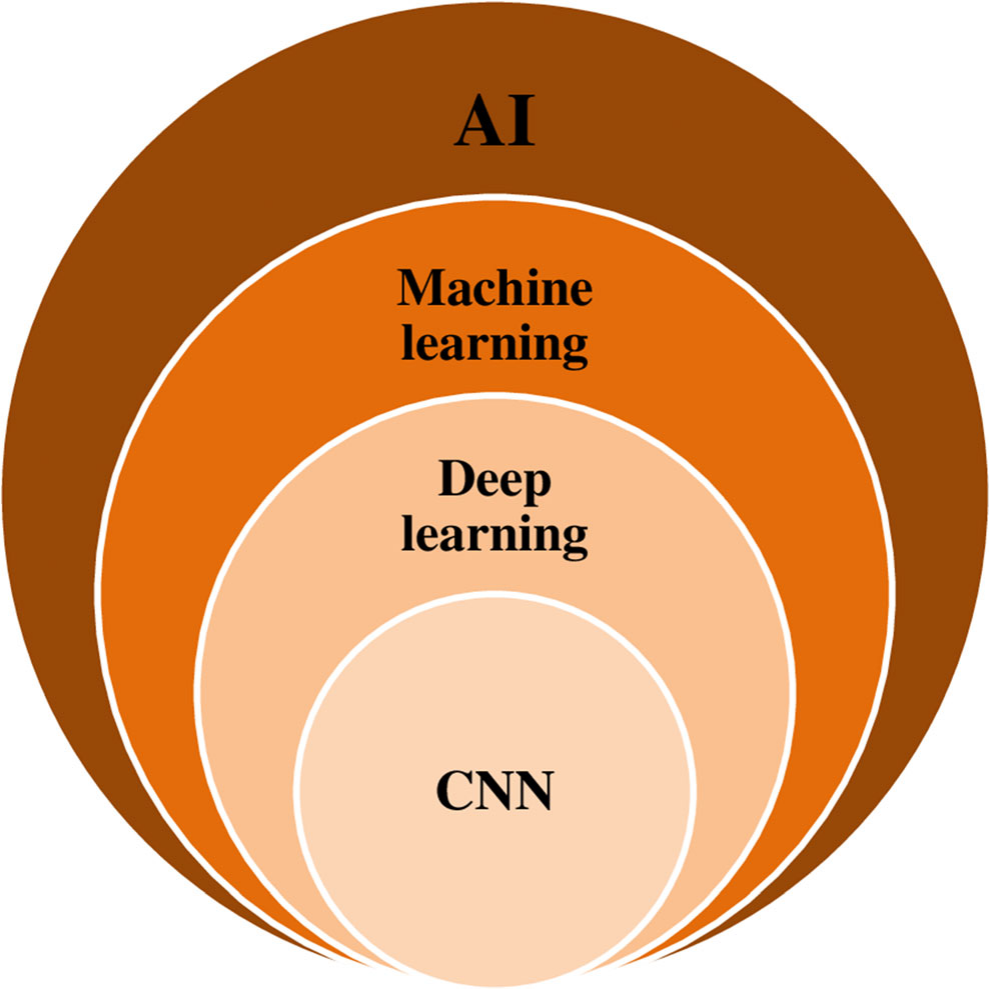
Artificial intelligence progression diagram (AI, artificial intelligence; CNN, convolutional neural network)

AI component’s virtual subclass is machine learning (Fig. 1), comprising mathematical algorithms used by computer systems in order to learn a specific task through experiences, without specific human instructions [6].

The advancement of that is deep learning (DL), consisting of a multi-layer representation learning architecture. The representation activates the first layer of neurons through a sensor, which, in turn, activates the next layer by complex connections. Each layer processes the representation in a non-linear way, creating an increasingly complex schema, diverging from the general machine learning task-specific algorithm [7, 8].

DL’s major advantage is that it can improve autonomously, without human input. From a usage standpoint, it can perform arbitrary parallel computation more efficiently than other algorithms [9, 10]. DL is used in visual object recognition [7], speech recognition [11], driving assistance [12], and language classification [10] among others.

The first algorithm successfully used for pattern recognition was neocognitron, in 1980, which integrated neurophysiological architecture [8, 13, 14]. The key for successful feature extraction is in creating an appropriate network architecture, as shown by the apparition of backpropagation technique, in 1989, which allowed handwritten digit recognition, becoming a landmark reference [15].

CNNs require large, balanced datasets and advanced algorithms, reflecting into processing power and storage capacity requirements [8, 9]. Krizhevsky et al developed a CNN model named Alex Net by gathering the biggest database for training: 1.2 million images. The algorithm was able to classify the images into 1000 nature categories with the smallest possible error rate [16], making it the state-of-the-art database for training CNNs.

Performance wise, the first neural network to achieve superhuman performance in visual pattern recognition (http://people.idsia.ch/~juergen/superhumanpatternrecognition.html) appeared in 2011, when Ciresan et al used a deep neural network on graphics processing unit to recognize traffic signs images. In the last decade, graphics processing unit improvements made possible shorter computation time for complex operations in a common setting, flourishing CNN development [8, 17].

**Fig. 2:**
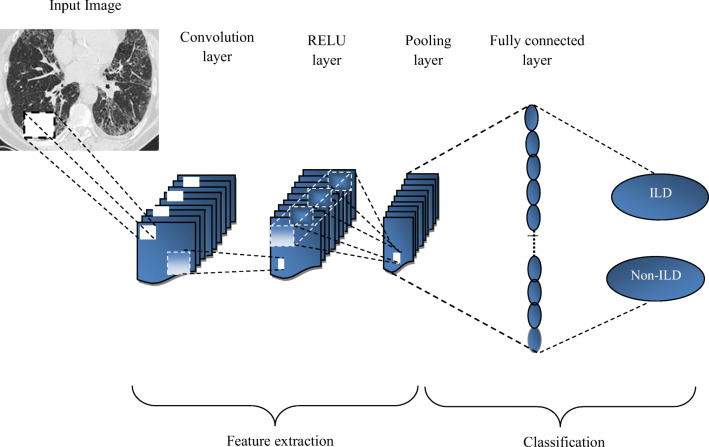
Convolutional neural network architecture

Each CNN has a complex architecture with an initial image input as pixel array from a receptive field with several hidden computational connection layers afterward [6, 7] as detailed in Fig. 2.

The convolutional layer, CNN’s main component, consists of multiple, weighted individual filters [7]. The multiple filter sets detect different patterns in images. The detection progresses from small patterns such as corners, lines, and edges to shapes and objects.

For a CNN to perform, it requires multiple layer types and transmission between them [6]. The final stage predicts the image category probabilities, determining the strongest and the most relevant active feature class [16].

The earliest CNN application in the Healthcare field dates into the early 1990s. Lo et al applied the CNN algorithm for lung nodule detection on chest X-rays, reaching a true-positive detection rate of 80% [18]. Sahiner et al used CNNs to classify mass from normal breast tissue on mammograms, with a 90% positive value [19]. In 2008, CNNs successfully detected hippocampal sclerosis on brain MRI [20]. These first studies’ high positive rates are biased due to the small database and easily detected lesion types.

There are multiple challenges in acquiring medical images for using in deep learning:

(i) They are difficult and costly to obtain, compared with common images.
(ii) To validly annotate bio-images, specialists must be used.
(iii) The medical database volume is generally insufficient, while the state-of-the-art image analysis datasets (ImageNet, AlexNet, GoogLeNet, VGGNet) contain thousands or even millions of natural image instances.

A workaround could be transfer-learning, where weights from a trained CNN on a nature dataset are conveyed to another CNN with a different dataset [6, 21].

Even though natural images and medical images are greatly different, the former being colored, whereas X-rays, CT images, and MRI images are gray-scaled, they all share the same descriptors. Histograms of oriented gradient and scale-invariant feature transform have been successfully applied to medical image segmentation and detection. Bar et al validated this in chest pathology by applying CNNs trained on non-medical image datasets to 93 chest X-ray images [22]. The area under the curve (AUC) was 0.93 for right pleural effusion detection, 0.89 for heart enlargement detection, and 0.79 for classifying normal versus abnormal chest X-rays. All values are well above 0.5, showing excellent model skill.

Starting in 2014, CNN applications in bio-imaging research flourished in segmentation, detection, and classification applications like lung nodule detection and classification, colon polyp detection, coronary calcification detection [23, 24], skin cancer classification [25], knee cartilage segmentation [26], brain tumor segmentation [27], and breast lesions classification [28, 29]. At present, the intention is to achieve higher accuracy and better performance than that of a human counterpart.

Although AI research has been predominantly directed to neurology [30], oncology, and cardiovascular diseases [6], as the first three major death causes, the chest imaging field also presents interest for lung nodule detection and classification [31, 32], tuberculosis lesion classification [33], lesion detection and classification [34], and parenchyma pulmonary disease classification [35].

## Interstitial lung disease–specific CAD

**Fig. 3:**
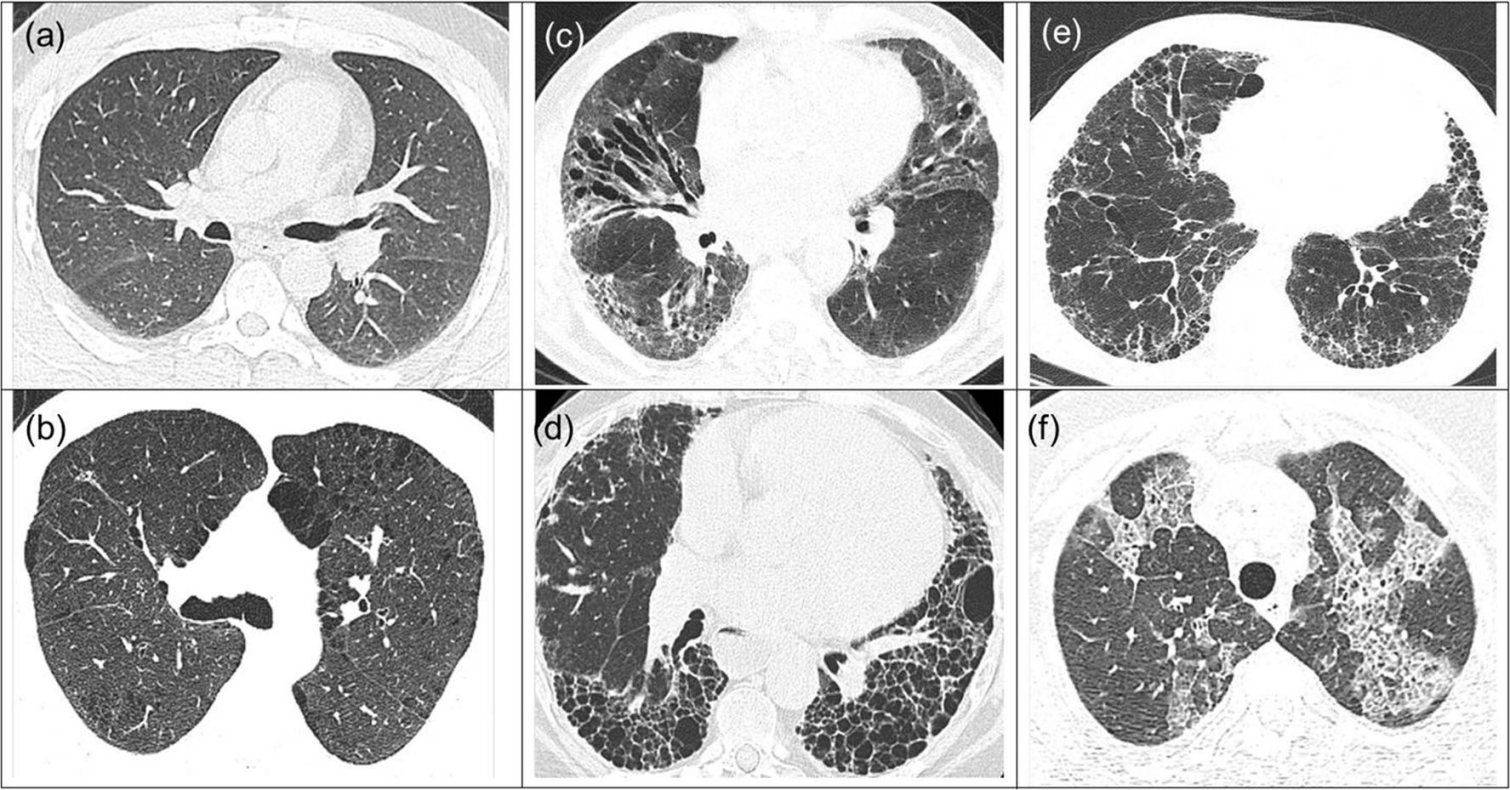
Difficult ILD patterns. **a** NL in subject 1. **b** EM in subject 2. **c** RE in subject 3. **d** HC in subject 4. **e** Mixed HC/RE in subject 5. **f** Mixed RE/GGO in subject 6. Source: “Victor Babes” Database

Typical ILD patterns in (HR) CT images are reticulation (RE), honeycombing (HC), ground-glass opacity (GGO), consolidation (CD), micro-nodules (MN), emphysema (EM), or combinations of the above. The difficulty appears when the results are combined or inconclusive (Fig. 3)

CNNs need large image samples because normal lung or different tissue categories could exhibit similar appearances (Fig. 3a, b or c, d), while significant variations might be seen between different subjects for the same tissue class (Fig. 3c–e).

Anthimopoulos M. et al [36] designed and trained one of the first CNN to classify the most common ILD patterns, achieving an 85.5% classification performance, therefore demonstrating the DL recognition capacity for lung tissue idiosyncrasy. Experienced radiologists annotated 120 HRCT by excluding ambivalent lung areas and the bronco-vascular tree which were used afterwards for training and testing the CNN. The proposed algorithm achieved superior performance compared with the state-of-the-art methods at that time (Alex Net, VGG-Net-D), mainly because of the hyper-parameters developed for the ILD pattern characterization. However, a misclassification rate was found between HC and RE due to their common fibrotic nature as shown in Annex 1 in the Supplementary Material. The combination between GGO/RE and individual GGO and RE pattern had also a high misclassification rate due to the overlapping appearance. Clinically, this reflects the significance in differentiating idiopathic pulmonary fibrosis (IPF) vs. non-specific interstitial pneumonia, as an accurate description of texture apart of gray-scale intensity value.

Dealing with these challenges, Christodoulidis S. et al [21] presented a CNN architecture that could extract the textural variability of ILD patterns. Using transfer learning from multiple non-medical source databases, they achieved an unsatisfactory increase of only 2% in the CNN performance. The big downfall in [21] is the usage of CT images, instead of HRCT.

Applications that use HRCT are only a few, like [37–39], all using CNNs for categorization. Even if Li et al [38] and Li et al [39] go a step further by using a custom architecture in an unsupervised algorithm, their performance is not as good as other options (e.g., [21, 40]).

### How complex the CNN should be?

Kim et al [40] compared shallow learning (SL) with DL in pattern classification. In their study, they used 4 convolutional layers and 2 fully connected layers for the CNN architecture that proved to have a significantly better accuracy from 81.27 to 95.12% by only increasing the number of convolutional layers. This lowered the misclassification rate between ambiguous cases such as HC/RE (Fig. 3e) or NL/EM and emphasized that a higher complexity of DL methods should be applied for a better ILD diagnostic.

Distinguishing between different lung tissue patterns on HRCT images is a challenging task, especially when using small samples in region of interest (ROI)–based classification. This could lead to mismatches since the lung tissue may have the same appearance between different tissue categories with great variation in the same category for different patients. Image processing consists not only in the gray-scale differences but also in object detection, regardless of variant parameters. Considering that patient movement during scanning and different types of breathing might affect lung volume size, Wang Q. et al [41] proposed a multi-scale rotation-invariant CNN algorithm to overcome this bias. This approach uses a Gabor filter, which analyzes the specific frequency and directions in a localized region, making it more like the human visual cortex. The performance accuracy achieved by this algorithm in classifying all the ILD patterns was greater than 85%, up to 90% for N, GGO, and MN patterns. The error rate has decreased by increasing the number of the CNN layers, similar to the previous study [40]. The downfall of this approach is the exponential complexity in the Gabor filter implementation, which requires significant resources to compute.

All other studies [21, 36, 40, 41] that focused on classifying ILD patterns employed a patch-based image representation method. Their pitfall consisted in the very small image sections (~ 31 × 31pixels), which resulted in fine detail loss. Furthermore, the image-patch needed to be manually annotated, creating an arduous process for radiologists.

By recognizing the impediment in manual identification of ROIs for automated pulmonary CAD systems, Gao M. et al [42] tried a different approach in ILD pattern classification. They proposed a holistic image recognition method, based on the gray-scale level, similar to emphysema quantification [43], but with greater autonomy. This perspective captured more details and used slice level image labels or tags without outlining the ILD regions. Rescaling the CT image in Hounsfield unit (HU), the method expresses three different attenuation scales in regard to the lung ILD pattern: low attenuation pattern (HU = − 1400 and − 950) such as EM, high attenuation pattern (HU = − 160 and 240) for CD, and normal lung attenuation (HU = − 1400 and 200). The holistic approach achieved 68.6% accuracy, less than the patch-based classification with 87.9% accuracy. However, the overall results are misleading: the holistic method classified EM perfectly; its difficulties were in separating normal lung (NL), MN, and CD pattern. The perfect EM classification warrants further exploration, maybe in a mixed method approach.

The dataset remains the Achilles heel in all of these approaches. An interesting approach was proposed by Bae HJ. et al [44], by making an infinite number of arbitrary different ILD patterns from 2D HRCT images which increased the accuracy of CNN in classifying lung tissue patterns. The algorithm prevented over-fitting, stabilizing accuracy loss for the validation set and providing a diverse mix of ILD patterns. Accuracy on specific region of interest or on the whole lung was 89.5%, higher than the conventional CNN data augmentation (82.1%), close to the human’s capacity. Best results were obtained for NL, GGO, RE, and EM. One of the algorithm’s drawback scan be randomization of ILD patterns which cannot mathematically insure a hypothetical perfect accuracy. Also, the iterative nature of the algorithm requires considerable computation resources, unfit for the normal computer.

### Idiopathic pulmonary fibrosis—the challenge of all ILDs

HRCT plays a central part both in the diagnosis and in management of all interstitial lung diseases, in particular in fibrotic lung disease. In an appropriate clinical context, idiopathic pulmonary fibrosis (IPF) diagnosis can be made without surgical lung biopsy, when the HRCT features are of usual interstitial pneumonia (UIP) [45]. Based on growing evidence, a statement from the Fleischner Society expanded this recommendation to include patients with features of probable UIP [46, 47]. Despite this framework, the ILD radiological assessment is still a challenge, due to substantial inter-observer variability, even between experienced radiologists [48, 49].

In clinical practice, this can be a challenge because imaging expertise is not always available, specifically in non-academic centers. This could lead to diagnosis delay and unnecessary interventional investigations, like surgical lung biopsy, which might pose unacceptable risks, especially for the older patients with advanced disease.

To overcome these limitations, Walsh et al [35] proposed a CAD that could easily be deployed on standard computing equipment.

A total of 1157 HRCT scans underwent a pre-processing step to create a maximum of 500 unique four-slice montages (concatenations) per CT scan resulting in a multiplied image dataset of 420,096 unique montages for the training algorithm and 40,490 for the validation set. The specific neural network architecture used in this study was the convolutional neural network Inception-ResNet-v2 [50, 51]. Each HRCT was classified by an experienced thoracic radiologist in one of the three categories: UIP, possible UIP, or inconsistent with UIP [45], with the specific diagnostic prediction outcome. A specific optimization algorithm was used to adjust the network’s internal parameters and to reduce the scan errors, making the neural network training an interactive process. The algorithm accuracy was 76.4%, tested on 139 HRCT (68,093 unique test montages), with 92.7% of diagnoses within one category. The algorithm needed 2.31 s to evaluate 150 four-slice montages.

This algorithm [35] was clinically tested on a second 150 HRCT scan cohort with fibrotic lung diseases. Numerous patients with a multidisciplinary team diagnosis of IPF, chronic fibrotic hypersensitivity pneumonitis, or connective tissue disease–related fibrotic interstitial lung disease were evaluated by 91 thoracic radiologists (not involved in the training process). The performance against the radiologists’ opinion was an average of 73.3% (93.3% within one category). The median accuracy of the thoracic radiologists on this cohort was 70.7%. By providing reproducible, almost instantaneous reporting with human-level accuracy, this algorithm stands as an important diagnosing tool for IPF.

Since the UIP pattern is known to be related to high mortality rates in ILD, the labeling of UIP vs. non-UIP is very important. The algorithm and the radiologists’ majority opinion provided equally prognostic discrimination (*p* = 0.62) between these two groups [35]. When Fleischner criteria for IPF diagnostic were taken into consideration, a good inter-observer agreement between algorithm and the radiologists was noticed. Although CNN is not trained to recognize basal honeycombing as a distinguishing feature of UIP, it seems that it can learn how to recognize it. This autonomous behavior might provide a framework for discovery novel image biomarker in fibrotic lung disease. The problem with this algorithm is that it considers only one tissue subtype in one slice, eliminating all the mixed patterns. The huge advantage, however, is its availability and low local resource requirements.

## Discussion

One major conundrum in developing high-accuracy deep learning algorithms for fibrotic lung disease diagnostics is the lack of large imaging datasets for training. To overcome this problem, a centralized imaging repository needs to be created through an international collaborative effort. Not only that, but the images need to be unified under a desirable format, HRCT. There are too few algorithms that deal with these image types like [35, 37–39] and since the 1-mm-thick slices can show lesions otherwise omitted, this format is a necessity. Since the images will, more than likely, be gathered from multiple sources, the resolution, gray-scale, and annotations need a unified format, also.

A handle-able CNN that could be deployed on any computer station and accessible to non-academic centers is the “Next Generation” of AI in clinical practice. Namely, in the field of ILDs, AI can help to differentiate and early diagnose the patients with the most severe forms, i.e., IPF. Early diagnosis in IPF will lead to targeted antifibrotic treatment which substantially prolongs the survival and reduces acute exacerbations which are not only deadly but also costly [52–56]. In order to reach such high expectations, a hybrid algorithm should be developed. Since some algorithms show exquisite accuracy in some areas, like [42], a combination of multiple CNN can be the answer to reducing the costs spent on the social and healthcare aspects [57, 58]. The CNN combination could have different configurations and start forming the same input or, on the contrary, have similar structures and different inputs. Even more interesting would be a combination of different AI techniques, like CNN with clusterization and classification algorithms, maybe in different stages, parallel or subsequent. No matter what the approach is, the purpose is to keep local area computing to a minimum, leaning therefore towards a cloud architecture style. In this case, legal aspects, like data privacy and security, begin to play an important role and the communication between the local and computational nodes could be obstructed by security protocols.

## Conclusions

In this review, we describe the deep learning algorithm developments and their implication in the medical field, especially in the ILD diagnostic. We highlighted the challenges, but also the implementation options that would, one day, lead to daily practice, with a clinical implication in early diagnosis of ILDs. Developing a CAD that could be deployed on any computer station and be accessible to non-academic centers is the next frontier in the early diagnosis of IPF.

## Electronic supplementary material

ESM 1 (DOCX 19 kb)

## Abbreviations

AI: Artificial intelligence
AUC: Area under the curve
CAD: Computer-aided diagnosis
CD: Consolidation
CNN: Convolutional neuronal network
CT: Computer tomography
DL: Deep learning
EM: Emphysema
GGO: Ground-glass opacity
HC: Honeycombing
HRCT: High-resolution computed tomography
HU: Hounsfield unit
ILD: Interstitial lung diseases
IPF: Idiopathic pulmonary fibrosis
IT: Information technology
ML: Machine learning
MN: Micro-nodules
MRI: Magnetic resonance imaging
NL: Normal lung
RE: Reticulation
ROI: Region of interest
UIP: Usual interstitial pneumonia
