# Supplementary material for: Deep learning in interstitial lung disease—how long until daily practice
Source: Eur Radiol. 2020 Jun 14;30(11):6285–92. doi: 10.1007/s00330-020-06986-4 (PMC7554005; doi:10.1007/s00330-020-06986-4)
Supplement: Supplementary file 1 — (DOCX 19 kb) [file 330_2020_6986_MOESM1_ESM.docx]

| **Study** | **Database** | **Algorithm** | **ILD pattern** | | | | | | | | | | | | **Particular aspects** |
| --- | --- | --- | --- | --- | --- | --- | --- | --- | --- | --- | --- | --- | --- | --- | --- |
|  |  |  | NL | EM | HC | RE/  GGO | CD | RE | GGO | | FB | | MN | |  |
| Anthimopoulos et al.  2015 | - 120 CT scans from different scanners and hospitals | - CNN (5 conv layers) | 0.99% AUC |  | 0.96%AUC | 0.95%AUC | 0.99%AUC | 0.96%AUC | 0.98%AUC | |  | | 0.99%AUC | | - use of 2x2 kernels, Leaky ReLU  - Adam, a first-order gradient-based algorithm |
| Christodoulidis et al.  2016 | - source domains: 6 texture benchmark databases  - target domain: 109 HRCT | - CNN (5 conv layers)  -Single-source transfer learning  - Knowledge fusion in an ensemble  -Model compression  -Multi-task learning |  | | | | | | | | | | | | - single source learning  - multi-task learning as a baseline method  - knowledge fusion in an ensemble  - CNN compression |
|  |  |  | 0.855%F avg | | | | | | | | | | | |  |
|  |  |  | 0.8817%F avg | | | | | | | | | | | |  |
|  |  |  | 0.8751%F avg | | | | | | | | | | | |  |
|  |  |  | 0.8631%F avg | | | | | | | | | | | |  |
| Gao et al.  2016 | - 120 HRCT from publicly available ILD database | -CNN (5 conv layers) | 0.40%AUC | 1.00%AUC |  |  | 0.50%AUC |  | 0.75%AUC | | 0.80%AUC | | 0.56%AUC | | - CT attenuation rescale, 3 attenuation scales concerning to lung abnormality patterns  - data augmentation  - patch base classification vs. holistic image classification |
| Wang et al.  2017 | -113 sets of HRCT from publicly available ILD database | -MRCNN (3 conv layers) | Gabor LBP= 90.1 % AUC  Gabor = 89.7% AUC  LBP = 86.7% AUC | | | | | | | | | | | | - multi-scale CNN  - rotation-invariant Gabor LBP images  - changing the overlapping  size of adjacent patches |
| Kim et al.  2017 | -HRCT from 106 patients, Siemens (S) CT scanner  -HRCT from 212 patients,  General Electric (GE) CT scanner | -CNN (4 conv layers)  -SVM | Intra-scanner G = 96.06 ± 1.61% AUC  Intra-scanner S = 96.11 ± 1.19 % AUC  Integrated scanner = 95.12 ± 1.91 % AUC  Inter-scanner G2S = 86.13 ± 2.28 % AUC  Inter-scanner S2G = 85.04 ± 1.91% AUC | | | | | | | | | | | | - comparison of shallow and deep learning methods for intra-scanner, inter-scanner, and integrated scanner data  -data augmentation employed to CNN |
| Bae et al.  2018 | -HRCT from 106 patients | -FusionNet CNN | 98.9% AUC | 90.5% AUC | 81.8% AUC |  | 91.6 % AUC | 84.3% AUC | | 90.0% AUC | |  | |  | -CNN with Perlin noise data augmentation vs. CNN with conventional data |
| Walsh et al.  2018 | -1157 anonymous HRCT scans from two IPF institutions | -CNN | UIP or not UIP  79.3% specificity  90.1% sensitivity  Inconsistent UIP or not inconsistent UIP  84.7% sensitivity  74.6% specificity  Possible UIP or not possible UIP  33.3% sensitivity  89.2% specificity | | | | | | | | | | | | - image pre-processing and resampling in 4 slice combinations converted into image montages  - assessment of cases using the Fleischner criteria |

Annex 1. Deep learning for classifying fibrotic lung disease on high-resolution computed tomography (HRCT). ILD patterns: normal lung (NL), emphysema (EM), honeycombing (HC), reticulation (RE), ground glass (GGO), consolidation (CD), fibrosis (FB), micronodules (MN). CNN’s area under the curve as percentages (%AUC). Performance and comparison between CNN methods expressed as Average Function Value (F avg).

<https://drive.google.com/open?id=1_2bEdecQlYuks76HfZZdeikhy_dCERxr> <http://tiny.cc/08p4iz>
